# Understanding malaria resurgence in the Amhara Region, northwestern Ethiopia: a qualitative study of perceived drivers from stakeholder and community perspectives

**DOI:** 10.1186/s12879-026-12929-z

**Published:** 2026-02-19

**Authors:** Mastewal Worku Lake, Kassahun Alemu Gelaye, Mulusew Andualem Asemahagn, Kindie Fentahun Muchie, Muluken Azage Yenesew

**Affiliations:** 1 Department of Epidemiology and Biostatistics, School of Public Health, College of Medicine and Health Sciences, Bahir Dar University, Bahir Dar, Ethiopia; 2 Amhara Public Health Institute, Bahir Dar, Ethiopia; 3 Department of Epidemiology and Biostatistics, Institute of Public Health, University of Gondar, Gondar, Ethiopia; 4 Department of Health Systems Management and Health Economics, School of Public Health, College of Medicine and Health Sciences, Bahir Dar University, Bahir Dar, Ethiopia; 5 Department of Environmental Health, School of Public Health, College of Medicine and Health Sciences, Bahir Dar University, Bahir Dar, Ethiopia

**Keywords:** Malaria, Resurgence, Health systems, Qualitative research, Community perceptions, Vector control, Conflict, Ethiopia

## Abstract

**Background:**

Ethiopia has experienced a marked resurgence of malaria, with confirmed cases rising from 1.0 million in 2018 to more than 7.3 million by October 2024. This resurgence is particularly pronounced in the Amhara Region and threatens national elimination goals. This study explored perceived drivers and contextual determinants of malaria resurgence from the perspectives of frontline health workers and community members in the Amhara Region.

**Methods:**

Between 1 and 28 February 2025, we conducted 28 semi-structured key informant interviews, two focus group discussions (*n* = 20), and 12 in-depth interviews. Participants were purposively selected based on professional roles or community leadership. Data were collected in Amharic, audio recorded, transcribed verbatim, and translated into English, then analyzed inductively using reflexive thematic analysis. The Health Belief Model, which focuses on risk perception and care-seeking behavior, was used as a sensitizing framework to interpret behavioral findings.

**Results:**

Participants perceived malaria resurgence as increasingly widespread, with near year-round transmission, expansion into previously low-risk highland areas, and a rising burden among children under five years and pregnant women. Perceived ecological drivers included irregular rainfall, land use change, irrigation schemes, and construction-related water storage. Reported health system challenges included recurrent stockouts of antimalarials and diagnostics, delayed or incomplete vector control, and reliance on passive surveillance. Behavioral factors such as low perceived susceptibility and severity, reliance on traditional and religious healers, misuse of insecticide-treated nets, and weak regulation of private providers were seen to delay care-seeking and undermine prevention, particularly in conflict-affected and remote areas.

**Conclusions:**

Stakeholders and community members perceived malaria resurgence in the Amhara Region as arising from interacting ecological change, health system fragility, and behavioral dynamics, exacerbated by conflict and recent public health emergencies. These hypothesis-generating findings suggest that locally tailored, equity-focused interventions, integrating behavioral insights with strengthened supply chains, surveillance, and community-centered vector control, could inform efforts to address resurgence and support Ethiopia’s elimination goals.

**Supplementary Information:**

The online version contains supplementary material available at 10.1186/s12879-026-12929-z.

## Introduction

Malaria remains a major cause of morbidity and mortality in tropical and subtropical regions globally [1]. In 2023, an estimated 263 million malaria cases and 597,000 deaths occurred across 83 endemic countries, with the WHO African Region accounting for 94% of cases (246 million) and 95% of deaths (569,000) [2]. Despite the scale-up of vector control interventions, such as insecticide-treated nets (ITNs) and indoor residual spraying (IRS), and the availability of effective antimalarial therapies, global malaria control remains fragile, and progress has stalled in many settings, necessitating sustained investment and adaptive strategies [3, 4]. In several endemic countries, including Ethiopia, these gains are now being threatened by a resurgence of malaria. Weakening health systems, reduced funding, and declining coverage of ITNs and IRS have been linked to renewed transmission in multiple contexts [3, 5].

The dynamics of resurgence are clearly visible in Ethiopia. Following the scale-up of key interventions after 2005, reported malaria cases declined from 5.2 million in 2004 to approximately 1.0 million in 2018, an 80% reduction [6]. However, this trend has reversed: by 20 October 2024, more than 7.3 million cases had been reported nationally, the highest annual figure in recent years [2]. Northwestern Ethiopia has historically experienced high malaria prevalence, and recent surveillance indicates increasing case numbers in several districts [7–9]. Malaria remains endemic in the Amhara Region of Ethiopia, especially in its western lowland areas where altitudes are below 2,000 m above sea level [10–12]. The region contributes a substantial proportion of Ethiopia’s malaria burden, often accounting for around one third of national cases [2, 13].

Multiple factors have been implicated in malaria resurgence globally, including insecticide and antimalarial drug resistance, climatic and ecological changes, population movement, and health-system fragility. Socio-political crises and declining funding further increase vulnerability in endemic regions [2, 5, 14]. Recent trends highlight emerging threats, such as the spread of artemisinin resistance in Africa [15] and the re-emergence of imported cases in countries approaching elimination, including Cambodia and Bhutan [16]. Molecular surveillance in East Africa, including Ethiopia, has identified nonsynonymous kelch13 mutations (e.g., R622I, C580Y) that may be associated with delayed parasite clearance, although their operational significance is not yet fully characterized [17, 18].

In Ethiopia, two contemporary threats are particularly relevant to the observed resurgence in the Amhara Region: the disruptive impact of armed conflict and the spread of the invasive vector *Anopheles stephensi.* Ongoing conflict has destabilized parts of northwestern Ethiopia, leading to the displacement of populations, disruption of supply chains, and diversion of health budgets, severely compromising the continuous delivery of essential malaria services. Furthermore, the identification of *A. stephensi* in the region presents a novel challenge, as this mosquito species thrives in urban environments and exhibits high insecticide resistance, potentially undermining traditional control efforts and driving urban transmission cycles [5, 14]. Understanding how these emerging systemic and biological threats intersect with local perceptions is crucial.

Behavioural and perceptual factors also play a central role in shaping malaria prevention and care-seeking. Health beliefs, perceived susceptibility, and treatment-seeking norms influence the uptake and sustained use of interventions. These constructs align with the Health Belief Model (HBM), which offers a useful lens for interpreting community risk perceptions and health behaviours in malaria-endemic contexts, and was therefore used as a sensitising framework in our interpretation [19]. In settings experiencing resurgence, low risk perception and substantial barriers to care can contribute to delays in seeking treatment and poor adherence to preventive measures [20].

While quantitative surveillance data are essential for tracking trends and evaluating programmes, they do not fully capture the lived experiences that shape how communities and frontline workers understand and respond to malaria. This represents a critical knowledge gap, particularly in regions experiencing resurgence. We therefore adopted a qualitative, phenomenologically informed approach to explore how experts, frontline health workers, and community members perceive and interpret malaria resurgence in the Amhara Region. Unlike prior studies focused on single districts or programmatic actors, this study integrates expert, frontline, and community perspectives across ecological zones in a conflict-affected region. By centering their voices, this study aims to identify systemic and behavioural barriers and generate context-specific insights to inform resilient malaria control strategies [21].

## Methods

### Study design and period

We used an interpretivist, phenomenologically informed qualitative design focused on participants’ lived experiences and meanings related to malaria resurgence and conducted reflexive thematic analysis as described by Braun and Clarke [22]. The study was conducted from February 1 to 28, 2025.

### Study setting

The Amhara Region is located in northwestern Ethiopia (9°20′–14°20′N, 36°20′–40°20′E) and has an estimated population of about 25 million [23]. Rainfall patterns vary across the region, with some zones receiving 75–80% of annual rainfall over five to six months, compared to about four months in others [24]. The region’s mean annual maximum and minimum temperatures are 25.8 °C and 12.6 °C, respectively, with peak rainfall occurring from June to August [25]. Ethiopia is administratively divided into regions, zones, woredas (districts), and kebeles (the smallest administrative units, comparable to wards or neighbourhoods).

We purposively selected woredas from both highland (Dega) and lowland (Kolla) ecological zones and areas with recent increases in malaria caseloads. Within these woredas, health facility catchment areas were selected to ensure representation from both rural and urban kebeles. In total, nine facilities were included: three hospitals, three health centres, and three private clinics. The full list of included woredas and facilities is provided in Additional file 1.

### Study population, sampling, and recruitment

We used a multi-stage purposive sampling strategy to maximise ecological and programmatic variation. First, woredas were selected to capture highland and lowland ecological zones and areas reporting recent increases in malaria cases. Within each woreda, health facilities (hospitals, health centres, private clinics) and their catchment kebeles were purposively sampled.

Professional participants (malaria experts, programme coordinators, clinicians) were recruited via existing professional networks and through implementing partners. Malaria experts were individuals with technical or leadership roles in malaria programmes at national, regional, zonal, or woreda levels or within implementing/partner organizations. These included epidemiologists, entomologists/vector control officers, surveillance and monitoring and evaluation officers, laboratory specialists, and programme managers; clinicians with malaria leadership responsibilities were also included. Malaria experts participated both in the dedicated expert focus group discussion (FGD) and, at regional and zonal levels, as key informants in key informant interviews (KIIs). National-level experts were primarily engaged via one FGD conducted alongside a scheduled regional meeting.

Community in-depth interviews (IDIs) participants (community leaders, volunteers, association members) were identified through the woreda administrative structures to ensure representation of women and marginalised groups. We conducted IDIs, rather than FGDs, with community leaders and volunteers to minimise power dynamics and reduce social desirability bias that might be heightened in group settings. Inclusion criteria were active involvement in malaria prevention, surveillance, or response in the Amhara Region within the preceding year. Individuals without direct programme or community engagement were excluded.

In total, 60 individuals were approached and screened; all were eligible and consented to participate (100% participation). No potential participants refused or withdrew from the study. This high participation likely reflects existing professional relationships and local support for the topic.

Data collection and analysis proceeded iteratively until thematic saturation was reached [26]. Saturation was assessed separately for KIIs, FGDs, and IDIs through ongoing review of coded transcripts. Saturation was defined as the point at which (i) no new codes emerged in later transcripts, (ii) increasing redundancy was observed in how participants described key concepts (“meaning saturation”), and (iii) informal saturation grids confirmed no substantive new insights within each participant group. Three researchers independently coded the first five transcripts to develop a preliminary codebook. Thereafter, all transcripts were double-coded, and discrepancies were resolved by consensus, a recognised approach to determining sample adequacy in qualitative research [26].

### Reflexivity

The research team comprised public health and epidemiology professionals with prior experience in malaria programme in Ethiopia. Trained qualitative researchers conducted interviews and FGDs. Reflexive discussions held before and throughout data collection and analysis addressed potential assumptions and biases. Detailed reflexive notes were maintained to ensure transparency in interpretation.

### Analytic framing and roles of data sources

We did not generate separate theme sets for each data collection modality. Instead, FGDs, KIIs, and IDIs were treated as a single, integrated dataset for coding and theme development. Initial codes were developed from a purposive mix of transcripts from all three sources. Themes were iteratively refined through constant comparison until analytic saturation was reached. In the Results, we present themes first and then illustrate convergence and divergence among experts, facility staff, and community participants. No single data source was privileged during coding or theme generation. Although routine surveillance figures are cited in the introduction to contextualise national/regional trends, we did not conduct a quantitative analysis or mixed-methods statistical integration in this study.

### Data collection tools and procedures

Two FGDs were conducted with a total of 20 participants. FGD 1 included ten malaria experts from the national malaria programme (with experience in Amhara Region), and from regional, zonal, and woreda-level programme and partner organisations. FGD 2 comprised ten clinical officers from health centres, hospitals, and private clinics. The expert FGD format was chosen to surface consensus and contestation, observe real-time prioritisation across domains, and complement role-specific technical detail captured in individual KIIs. Discussions were held in private meeting rooms at centrally accessible locations and were facilitated by a trained researcher, supported by a note-taker. No non-participants were present. Sessions lasted 55–75 min (mean 65 min), and no repeat FGDs were undertaken.

FGDs and KIIs were guided by semi-structured tools developed with reference to the WHO malaria programme guidelines and relevant literature [5, 27–30]. Core domains included recent trends and perceived drivers of resurgence; vector control and prevention activities (ITNs, IRS, and larval source management); diagnosis and case management; surveillance and reporting; preparedness and response capacity; community engagement and risk communication; environmental and climatic factors; and recommendations. The expert FGD and KII guides additionally explored policy, financing, intersectoral coordination, and supply chain governance, whereas the clinical officer tools included items on private-sector case management and resurgence-related workload. Detailed guides are provided in Additional files 2 and 3.

Twenty-eight face-to-face KIIs were conducted with regional, zonal, and woreda-level programme staff; clinicians from public health facilities; private healthcare providers; and implementing partner representatives. Regional and zonal experts participated in KIIs to gather detailed, role-specific technical information not easily obtained in group settings. KII were conducted by the principal investigator and a trained research assistant and lasted a mean of 40 min (range: 25–60 min).

We conducted 12 in-depth interviews (IDIs) with community leaders, volunteers, and members of local associations (such as *edir* [traditional mutual aid associations] and *ekub* [rotating savings groups]). Interviews were conducted in participants’ communities at locations of their choosing to enhance comfort and openness [31]. Two trained researchers conducted all IDIs, which lasted a mean of 35 min (range 25–50 min).

The IDI guide (Additional file 4) was developed using the same reference set as the FGD guide [5, 27–29], supplemented by constructs from the Health Belief Model. Domains addressed household experience and impact; perceived causes and seasonality; access to and use of ITNs and IRS; information sources and misconceptions; environmental exposures; care-seeking pathways; community-led initiatives; behavioural facilitators and barriers; and recommendations. Prompts based on perceived susceptibility, severity, benefits, barriers, cues to action, and self-efficacy informed the structure of the guide.

### Data recording and management

All FGDs, KIIs, and IDIs were conducted in Amharic, audio-recorded with participant consent, and transcribed verbatim. Transcripts were checked for accuracy against the audio recordings. There was no overlap of individuals across the three data collection modalities. No interviews or FGDs were lost or excluded after transcription; minor translation discrepancies were resolved during reconciliation. Topic guides are provided in Additional files 2–4.

### Data analysis

Data were analysed following Braun and Clarke’s six-phase thematic framework [21, 22]. All interviews and FGDs were transcribed verbatim in Amharic. Two independent professional translators produced contextualised English translations. To ensure accuracy, 10% of translated transcripts (*n* = 6) were back-translated into Amharic by a third professional translator and compared with the originals; minor discrepancies were resolved by consensus among the translators and the research team.

Three researchers independently coded the first five transcripts to develop an initial codebook. Thereafter, each transcript was double-coded by two researchers using ATLAS.ti version 9.0. Coding disagreements were resolved through iterative discussion between the two primary coders until consensus was achieved; when disagreements persisted, a third researcher was consulted to facilitate resolution. We did not calculate a numeric inter-coder reliability coefficient, as our analytic focus prioritized conceptual agreement and shared understanding of codes over statistical concordance. Emergent themes were visualised using a concept map to explore relationships between themes [32]. While the analysis was primarily inductive, we subsequently used the Health Belief Model as a sensitising framework to interpret behavioural themes in the Results and Discussion. HBM was applied during interpretation, not code generation. Because this study was qualitative and did not involve a quantitative dataset, procedures for handling missing data were not applicable.

The study adhered to the COREQ (Consolidated Criteria for Reporting Qualitative Research) 32-item checklist (Additional file 5).

### Trustworthiness

We applied Lincoln and Guba’s criteria of credibility, dependability, confirmability, transferability, and authenticity to enhance trustworthiness [33]. Credibility was supported through purposive sampling, triangulation of data sources (experts, health workers, community leaders), and in‑depth probing during interviews. Dependability was enhanced by using a multidisciplinary research team and a structured, consensus‑based coding process. Transferability was facilitated by detailed descriptions of the study context, sampling, and procedures. Confirmability was strengthened through documentation of analytic decisions, cross‑validation of translations, and grounding of interpretations in participant quotations.

## Results

### Sociodemographic characteristics of participants

A total of 60 participants were included in this study: 28 key informant interviews (KIIs), two focus group discussions (FGDs; *n* = 20), and 12 in-depth interviews (IDIs). Sociodemographic characteristics of FGD participants are summarized in Table 1. Among FGD participants, most were male and worked in government, NGO/partner, or private sectors; most had ≥ 5 years of malaria-related experience.

Among the 28 key informants, 17 (60.7%) were male, and 11 (39.3%) were female. The majority (18/28, 64.3%) were aged ≤ 49 years. Half (14/28, 50.0%) held a bachelor’s degree, nine (32.1%) had a master’s degree or higher, and five (17.9%) had a diploma. Most worked in the government sector (16/28, 57.1%), with additional representation from NGO/partner organisations (6/28, 21.4%) and the private sector (6/28, 21.4%). Twenty-four key informants (85.7%) had more than five years of experience in malaria programmeming or related fields.

Twelve participants were engaged in IDIs, most of whom were female (8/12, 66.7%). Seven (58.3%) were aged ≤ 49 years. Educational backgrounds varied: five (41.7%) had no formal education, four (33.3%) had a master’s degree or higher, and three (25.0%) held a bachelor’s degree. Participants were drawn from the private sector (5/12, 41.7%), community members without formal employment (5/12, 41.7%), and government (2/12, 16.7%). These IDIs primarily captured the perspectives of community members and programme beneficiaries, complementing the views of professional stakeholders obtained through KIIs and FGDs.

**Table 1:** Sociodemographic characteristics of focus group discussion participants (*N* = 20)

Characteristic	Category	*n* (%)
Sex	Male	13 (65.0)
	Female	7 (35.0)
Age group (years)	≤ 49	10 (50.0)
	≥ 50	10 (50.0)
Educational level	Diploma	7 (35.0)
	Bachelor’s degree	8 (40.0)
	Master’s degree or higher	5 (25.0)
Occupation/sector	Government	9 (45.0)
	NGO/partner organisation	6 (30.0)
	Private sector	5 (25.0)
Experience in malaria programme	< 5 years	5 (25.0)
	≥ 5 years	15 (75.0)

FGD, focus group discussion; NGO, non-governmental organization

### Overview of data sources and analytic approach

Themes were developed inductively across all data sources. In the Results, we organise findings by overarching theme; within each theme, we indicate points of convergence and divergence between (i) malaria experts, (ii) facility and programme staff, and (iii) community leaders and volunteers.

Five overarching themes were identified across all data sources: (1) environmental and climate drivers; (2) healthcare system gaps; (3) community engagement and perceptions; (4) external system shocks (armed Conflict and COVID-19); and (5) emerging biological threats (see Additional file 6 for thematic map). In the sections that follow, we describe each theme and illustrate how it manifested among expert, facility-based, and community participants.

### Perceptions of an escalating malaria burden

Experts described a marked resurgence of malaria nationally and in the Amhara Region, characterised by more sustained transmission and increasing burden among children under five years and pregnant women. They highlighted the spread into previously low-risk highland areas and a shift from seasonal to near year-round transmission.

The situation is worsening rapidly. Ethiopia registered over 7.3 million cases in 2024, which accounts for 3.6% of global malaria cases. (FGD‑expert, mixed gender)

Malaria cases in the Amhara Region have seen a radical increment across all ages, especially among children under five and pregnant women. This current trend marks a sharp contrast to the patterns observed in the past two to three decades. (KII- regional malaria expert, male, 38 years)

Health workers reported parallel trends in facility data, including sharp increases in cases and increased pressure on services during months that were previously considered low-transmission periods.

Malaria now persists even in dry months. Health facilities are being overwhelmed by Plasmodium falciparum and relapse cases associated with Plasmodium vivax. (KII-regional malaria expert, male, 42 years)

Community leaders echoed that malaria was “no longer seasonal”, describing multiple episodes per household throughout the year and frequent illness in children.

We get malaria so often now that people say it has become part of life. Even when it should be dry, children fall sick again and again. (IDI-community association leader, male, 38 years).

### Theme 1: Environmental and climate perceived driver

#### Expert perspectives

Experts consistently attributed the resurgence to climatic variability and environmental change, including irregular rainfall, rising temperatures, and unregulated development such as irrigation schemes and construction projects, which create new mosquito breeding sites.

Highland areas that were previously malaria-free are now reporting cases, likely linked to climate-driven temperature rises and irrigation projects. (FGD-experts, mixed gender)

Most experts related these local environmental changes to weak enforcement of national environmental and irrigation policies, noting that major projects rarely undergo systematic malaria risk assessment or mitigation planning.

#### Health worker perspectives

Health workers and programme staff described prolonged rains since around 2020, large-scale agricultural expansion with deforestation, and poorly managed irrigation that altered local ecologies and increased standing water.

Agricultural projects that are not integrated with malaria control efforts result in water-holding landscapes that support mosquito populations. (KII-woreda malaria focal person, male, 48 years)

#### Community perspectives

Community leaders provided concrete examples, particularly around urbanisation and construction. Most also noted that these developments proceeded without community consultation or apparent consideration of malaria risk, reflecting gaps in the application of national environmental and irrigation policies.

Uncovered water tanks at construction sites create new breeding grounds but remain unregulated. (IDI-community association leader, female, 28 years)

### Theme 2: Healthcare system gaps

#### Expert perspectives

Experts highlighted persistent health system weaknesses, including recurrent stockouts of antimalarial drugs (Coartem, primaquine), rapid diagnostic tests (RDTs), and delays or gaps in implementing IRS and ITN distribution. They also noted an over-reliance on passive surveillance.

Surveillance is mostly passive, leading to delayed case detection, while security and funding constraints limit active surveillance. (FGD-experts, mixed gender)

#### Health worker perspectives

Facility staff confirmed frequent disruptions in drug and supply availability and incomplete vector control coverage.

Coartem (artemether–lumefantrine) out of stock for four to five days each month, and chloroquine and primaquine were unavailable for six months. (KII-health officer, female, 34 years)

Only 3 out of 20 kebeles in our area received indoor residual spraying because of insecticide shortages. (KII-woreda malaria officer, male, 38 years)

Spraying was initiated at 168 cases per 1,000 population, but the national guideline recommends intervention at 50 cases per 1,000. (KII- regional malaria expert, male, 42 years)

Health workers also reported that stockouts in public facilities pushed patients toward private clinics, where prescribing practices were poorly regulated.

Public health facilities run out of drugs, pushing patients to costly private clinics. (FGD-clinical, mixed gender)

Some private clinics sell drugs based on patient demand, which can contribute to resistance. (KII- woreda malaria expert, female, 32 years)

Experts noted a decline in the contribution of health extension workers (HEWs) to malaria prevention activities.

The health extension programme’s contribution to malaria prevention has dropped to 20–30%, far below the expected 70%. (FGD-expert, mixed gender)

#### Community perspectives

Community interviewees reported the downstream effects of these system gaps.

Patients travel for hours to reach health facilities, only to find there is no medicine available. (IDI- community leader, male, 43 years)

### Theme 3: Community engagement and perceptions

#### Expert and health worker perspectives

Experts identified weak community engagement, low risk perception, and delays in care-seeking as critical barriers to malaria control. They described how severe symptoms, such as convulsions, were often attributed to spiritual causes, leading families to seek traditional or religious healers before formal care.

Severe symptoms like convulsions are attributed to spiritual causes, so families go first to traditional healers or holy water rather than the health facility. (FGD-clinical, mixed gender)

Experts and health workers also emphasised a gap between knowledge and practice.

People know malaria prevention methods but fail to implement them. (FGD-experts, mixed gender)

They noted a decline in proactive community-based work by HEWs, limited mass media communication, and underutilisation of influential community structures.

The health extension programme’s contribution to malaria prevention has dropped to 20–30%. (KII- national malaria expert, male, 39 years)

There are no mass media campaigns to reinforce prevention messages. Religious leaders, like priests, could help spread these messages. (FGD-clinical, mixed gender)

#### Community perspectives

IDIs illustrated how low perceived susceptibility, misconceptions, and practical constraints shaped behaviour, including misuse of ITNs and delayed care-seeking in hard-to-reach or conflict-affected areas.

Bed nets are used for storage, as a garden fence, or to make ‘gemed’ (rope)… not for prevention. (IDI- community volunteer, male, 35 years)

Communities often wait until they are very sick because travelling is difficult, and sometimes even health posts are closed. Access is a big challenge in conflict-affected areas. (IDI-community leader, male, 40 years)

I know malaria is around, but I don’t think I can get it. I keep my house clean, so I feel safe. (IDI- community leader, female, 42 years)

Participants also reported weak cues to action and low self-efficacy.

No one reminds us to drain stagnant water. (IDI-community leader, male, 44 years)

*“Mosquitoes everywhere overwhelm me; I feel helpless*,* unable to fight them off.”* (IDI-community leader, male, 41 years). These perceptions are summarised using the HBM in Additional file 7.

Across participant groups, these behaviours were framed as consequences of structural barriers such as stockouts, service disruptions, and insecurity rather than individual “failures,” highlighting the need to address both supply- and demand-side constraints.

### Theme 4: External system shocks (armed conflict and COVID-19)

#### Conflict-related disruptions

##### Expert and health worker perspectives

Experts identified armed conflict as a major external driver that disrupted malaria service delivery, supply chains, and funding. Health worker perspectives described health worker displacement, blocked roads, and budget diversion:

Ongoing conflict has displaced communities and diverted health budgets from malaria control to emergency responses. (KII-zonal malaria officer, male, 46 years)

Health extension workers have been displaced, and roads are blocked, making access to services nearly impossible. (KII-clinical health officer, male, 40 years)

Military checkpoints delay deliveries of Coartem by weeks. Patients die waiting. (KII- health officer, male, 33 years)

##### Community perspectives

Community leaders reported that closed health posts, long travel distances, and insecurity limited access to diagnosis and treatment, compounding the impact of the resurgence.

The COVID-19 pandemic represented a second major system shock that further undermined already fragile malaria services.

##### COVID-19-related *disruptions*

The COVID-19 pandemic further undermined malaria control:

During the pandemic, malaria was deprioritised, leading to a reduction in surveillance and control activities. (KII- woreda malaria officer, male, 38 years)

The effort to combat malaria was negatively impacted by COVID-19, which threatened to reverse gains towards elimination. (KII-clinical health officer, male, 38 years)

Participants described COVID-19 as one of the overlapping system shocks that, together with conflict, stretched already fragile services.

### Theme 5: Emerging biological threats

Our study did not include entomological sampling or molecular testing; the findings in this theme, therefore, reflect participants’ perceptions of possible drug and insecticide resistance and of *Anopheles stephensi*, rather than measured resistance or vector data.

#### Expert perspectives

Experts expressed concern about insecticide resistance and the invasive vector *Anopheles stephensi*, particularly in relation to urban transmission.

The invasive mosquito species Anopheles stephensi, which is highly reproductive and resistant to many insecticides, is perceived to be driving urban malaria transmission. (FGD-experts, mixed gender)

They also noted that mosquito densities appeared unchanged after IRS, raising suspicions about reduced insecticide effectiveness.

Mosquito density did not drop after IRS spraying, which implies possible resistance. (FGD-experts, mixed gender)

#### Health worker and community perspectives

Health workers reported apparent treatment failures and recurrent infections, while community members questioned the effectiveness of IRS.

Patients return with recurring infections despite completing treatment. (KII- woreda malaria officer, male, 32 years)

After they sprayed, we saw no change. The mosquitoes were just as wicked. We think the chemical no longer works. (IDI- community volunteer, male, 37 years)

### Application of the health belief model (HBM)

Behavioural findings from IDIs, supported by KIIs and FGDs, were interpreted using the HBM to understand community perceptions and care-seeking behaviours. HBM was applied during interpretation, not during initial code generation. A detailed table of illustrative quotations for each HBM construct is provided in Additional file 7.

## Discussion

This multi-level qualitative study adds new evidence on how ecological change, health system fragility, and community perceptions intersect to sustain malaria resurgence in the Amhara Region of northwestern Ethiopia. Unlike prior single-district or programme-focused studies, this research integrates expert, frontline, and community perspectives across diverse ecological zones in a conflict-affected setting underrepresented in malaria qualitative research. The findings are perceptual and hypothesis-generating; they do not establish causality or quantify the relative contributions of identified drivers to resurgence.

Participants reported that the resurgence disproportionately affects vulnerable groups, including pregnant women and children under five years, consistent with trends reported from other parts of sub-Saharan Africa during periods of climatic instability [3, 34]. Perceived increases in case numbers, a shift to near year-round transmission, and spread into highland areas mirror evidence linking ecological changes and climate variability to shifts in malaria transmission patterns [35, 36]. The NMESP 2021–2025 explicitly calls for intersectoral collaboration and environmental risk mitigation [12], yet our findings suggest these mechanisms are not yet operational at the woreda level. Operationalizing the NMESP’s intersectoral objectives will require mandatory malaria impact assessments for all large-scale irrigation and construction projects, enforceable water management standards, and routine environmental inspection.

Participants consistently linked resurgence to ecological changes, including irregular rainfall and unregulated development projects. This is in line with extensive evidence identifying landscape modification, such as irrigation, deforestation, and poorly managed construction, as key links to malaria transmission [37, 38]. The NMESP calls for intersectoral collaboration, yet our data suggest that large-scale agricultural and infrastructure projects are often not systematically integrated with malaria risk assessments or mitigation plans. Strengthening environmental governance, through mandatory malaria risk assessments for irrigation and construction projects, enforcement of water management standards, and regular environmental inspections, could operationalise the NMESP’s intersectoral objectives in concrete ways.

Participants emphasized that prolonged armed conflict has substantially disrupted routine malaria services in parts of Amhara. While our qualitative data cannot support regional comparisons or causal attribution, these perceptions underscore the urgent need for conflict-sensitive delivery models and emergency preparedness within the national elimination framework [12, 39]. The combined effects of conflict and COVID-19 resemble patterns documented in other fragile and conflict-affected settings, where overlapping shocks fragment health systems, block physical access, and erode trust, facilitating infectious disease resurgence [40–43].

Our findings on uncovered water tanks, urban construction sites, and improperly managed irrigation schemes resonate with studies from other parts of Ethiopia showing that such changes create sustained breeding sites and increase transmission risk [35, 37]. Concerns expressed about the invasive vector *Anopheles stephensi* and possible artemisinin resistance represent important field perceptions and early warning signals rather than confirmed phenomena. Given that *An. stephensi* has already been documented in other parts of Ethiopia and the Horn of Africa [43, 44]. These qualitative findings support the urgent need for targeted entomological surveillance.

Reports of apparent treatment failures and persistent mosquito densities after IRS should be interpreted as field observations that are suggestive of, but do not confirm, emerging molecular evidence of artemisinin partial resistance and insecticide resistance in Ethiopia [45–47]. Our study did not include therapeutic efficacy monitoring or entomological surveys; these perceptions require urgent empirical verification. The convergence between qualitative observation and molecular surveillance mirrors resistance emergence trajectories documented in Rwanda and Kigali [48]. A key recommendation from this study is to scale up targeted entomological surveillance and routine therapeutic efficacy studies to verify and characterise these concerns.

This study revealed persistent failures in supply chains, including recurrent stockouts of Coartem, primaquine, and RDTs, which undermined effective case management and pushed patients towards private facilities. Incomplete coverage of IRS and ITN distribution also undermines both case management and vector control. Such supply-chain challenges are common in resource-constrained settings where distribution systems are fragile [49]. Our findings on antimalarial and diagnostic shortages suggest structural weaknesses that have been exacerbated by political instability and COVID-19-related disruptions, similar to experiences in other conflict-affected settings such as South Sudan [50]. and in this context appear to have been exacerbated by political instability and COVID-19-related disruptions, similar to experiences in South Sudan and Yemen [40, 50]. These weaknesses directly challenge NMESP targets for universal access to diagnostics and effective treatment.

Behavioural barriers identified in this study can be systematically interpreted using the HBM [19]. Despite awareness of malaria, many community members reported low perceived susceptibility and severity, often viewing malaria as a routine, non-fatal illness. High perceived barriers, including stockouts, travel costs, and long waiting times, discouraged timely care-seeking and contributed to feelings of helplessness. Doubts about the benefits of IRS and ITNs, combined with weak cues to action (limited health education, absence of reminders), help explain persistent reliance on traditional healers and inconsistent use of preventive tools [51–53]. These findings reinforce the view that providing commodities alone is insufficient; programme must also address community perceptions, structural barriers, and social norms to improve uptake and sustained use of interventions [20]. They also highlight the need to revitalize the health extension programme and systematically engage community and religious leaders, as envisioned in the NMESP.

The collapse or weakening of core health-system functions, amplified by conflict and the COVID-19 pandemic, emerged as a central theme. Chronic stockouts, disrupted vector control, and a shift from active to largely passive surveillance are consistent with patterns documented in other conflict-affected and fragile settings, such as Yemen and South Sudan [40, 41]. Conflict not only diverts resources but also fragments health systems, blocks physical access, and erodes trust, all of which facilitate infectious disease resurgence [42, 54].

By integrating perspectives from experts, health workers, and communities, this study offers contextually grounded evidence on how local ecological changes, systemic weaknesses, and behavioural responses collectively sustain malaria transmission. These qualitative insights complement quantitative surveillance data and can inform the design of adaptive, locally responsive malaria control strategies. Policy efforts should prioritise rebuilding community engagement, strengthen supply chains and surveillance, and enhance programme resilience within a decentralised, resource-constrained system.

Future research should triangulate these perception-based findings with routine surveillance and environmental/climate datasets and prioritise (i) entomological monitoring to characterise insecticide resistance and vector composition, including mapping of An. stephensi where suspected; (ii) therapeutic efficacy studies and molecular surveillance to assess potential antimalarial resistance signals raised by frontline providers; and (iii) mixed-methods evaluations of intervention resilience in conflict-affected settings to validate and quantify these perceptual insights.

### Implications for policy and practice

Based on stakeholder and community perceptions, the following priorities could strengthen malaria control in the Amhara Region under Ethiopia’s NMESP 2021–2025:

1. Update malaria risk stratification to reflect transmission in highland and peri-urban areas, and integrate mandatory malaria risk assessments into environmental governance for irrigation, urban expansion, and large development projects.
2. Ensure reliable access to diagnostics and antimalarials through stabilized supply chains, buffer stocks in high-burden areas, and strengthened regulation of private providers.
3. Scale up social and behavioural interventions, including revitalization of health extension services, engagement of religious and community leaders, and targeted risk communication addressing low perceived susceptibility and structural barriers to care.
4. Establish routine monitoring of antimalarial drug efficacy, insecticide resistance, and Anopheles stephensi distribution to verify field-reported resistance concerns.
5. Develop flexible delivery models (mobile teams, community-based distribution, buffer stocks) for conflict-affected and hard-to-reach areas, and integrate malaria control into emergency preparedness and humanitarian response planning.

These recommendations are grounded in qualitative insights and should guide further empirical evaluation.

### Strengths and limitations

Strengths of this study include its multi-level perspective (from national experts to community leaders), purposive sampling across diverse ecological and programmatic settings, rigorous reflexive thematic analysis, and explicit attention to trustworthiness criteria. Triangulation across experts, health workers, and communities increased the credibility and depth of findings.

While credibility was enhanced through participant triangulation and adherence to Lincoln and Guba’s criteria [55], the study has limitations. Findings are based on stakeholder and community perceptions and may be influenced by recall bias, selective reporting, or social desirability, despite efforts to mitigate these through careful facilitation and confidentiality. The qualitative design does not allow causal inference or quantification of ecological, health system, or behavioural contributions to malaria resurgence; findings are hypothesis-generating. FGDs may have suppressed dissenting views, while IDIs may have limited capture of collective interactions; triangulation across methods helped address this. Themes were generated across pooled datasets, potentially obscuring modality-specific nuances, and purposive sampling may limit transferability. Finally, we did not triangulate findings with entomological, molecular, or routine surveillance data, nor collect detailed socioeconomic indicators; perceptions should be interpreted as field-level signals requiring further empirical investigation.

## Conclusion

This qualitative study integrated expert, frontline, and community perspectives to identify perceived drivers of malaria resurgence in the Amhara Region of Ethiopia. Findings highlight the interplay of ecological change, health system fragility, behavioural barriers, and external shocks (conflict, COVID-19) in sustaining transmission. Priority actions include operationalizing intersectoral environmental governance, stabilizing supply chains, revitalizing community engagement and health extension services, and building system resilience in conflict-affected areas. Future research should triangulate these perceptions with entomological surveillance (including Anopheles stephensi mapping), therapeutic efficacy studies, molecular resistance monitoring, and longitudinal epidemiology to verify and quantify these hypothesis-generating findings.

## Supplementary Information

Below is the link to the electronic supplementary material.

Supplementary Material 1

## Data Availability

De-identified data excerpts and the codebook are available from the corresponding author on reasonable request, subject to approval by the Bahir Dar University Institutional Review Board (Protocol No. 3053/2024). Full transcripts cannot be shared publicly due to confidentiality agreements with participants.
