# Supplementary material for: Understanding malaria resurgence in the Amhara Region, northwestern Ethiopia: a qualitative study of perceived drivers from stakeholder and community perspectives
Source: BMC Infect Dis. 2026 Feb 19;26:531. doi: 10.1186/s12879-026-12929-z (PMC12977728; doi:10.1186/s12879-026-12929-z)
Supplement: Supplementary file 1 — Supplementary Material 1 [file 12879_2026_12929_MOESM1_ESM.docx]

**Additional information**

Additional files (selected woredas, FGD, KII, and IDI guides; COREQ checklist; thematic map, and HBM findings) were listed

Additional file 1. Selected woredas by ecological zone in the Amhara Region, Ethiopia

| Ecological zone | Woreda/District |
| --- | --- |
| Highland (Dega) | Debre Tabor Town |
|  | Gaz Gibla |
|  | Lalibela Town |
|  | Mota Town |
|  | Lay Gayint |
|  | Debre Markos Town |
|  | Gondar Town |
|  | Dera |
| Lowland (Kolla) | Jabi Tehnan |
|  | Ayehu Guwagusa |
|  | Bahir Dar City |
|  | Adi Arkay |
|  | Baso Liben |
|  | Debre Elias |
|  | Chilga 2 |
|  | Kobo Town |
|  | Abergele (Amhara) |
|  | Shewa Robit |

*Note: Districts were purposively selected based on ecological classification and recent malaria trends.*

Additional file 2. Focus group discussion (FGD) guide for malaria experts and clinical health workers

| Section/topic | Main question | Probes (where applicable) | Purpose/notes |
| --- | --- | --- | --- |
| Introduction | — | Introduce facilitator and note taker; explain study purpose, confidentiality, anonymity, voluntary participation; audio recording and duration; ground rules; obtain informed consent | Set context, build rapport, ensure ethical conduct |
| 1. General understanding of malaria | What do people in your area know about malaria? | Transmission, common symptoms, perceived severity; who is most at risk | Explore baseline knowledge and perceptions |
| 2. Trends in malaria prevalence and resurgence | How has the burden of malaria changed in your zone/woreda/facility over recent years? What are the major factors contributing to resurgence? | Increasing/decreasing/fluctuating/resurgence; seasonality versus year‑round transmission; differences by age, sex, pregnancy, location; socioeconomic, environmental/climatic, health system, population movement factors | Characterise perceived trends, affected groups, and linked to |
| 3. Malaria prevention practices and coverage | What preventive measures are used to avoid malaria? How accessible and acceptable are these methods? | ITNs, IRS, larval source management; personal protection; housing improvements; coverage and use among general and vulnerable groups; gaps and inequities | Assess prevention practices, access, and acceptability |
|  | What challenges affect vector control implementation? Are community-based groups contributing to prevention? | Logistics/supply; community acceptance/misconceptions; operational constraints; roles of *edir*, *ekub*, women’s and youth groups; perceived effectiveness | Identify implementation barriers and community assets |
| 4. Diagnosis and case management | What challenges exist in diagnosing malaria cases? How effective are current treatment protocols? | Availability/quality of RDTs and microscopy; staff skills and workload; referral pathways; adherence to national guidelines; perceived treatment failures | Explore diagnostic capacity and quality of care |
|  | Are resources (drugs, diagnostics, supplies) sufficient? | Frequency/duration of stockouts; impact on care; mitigation strategies | Examine supply adequacy and reliability |
| 5. Surveillance and reporting | How effectively does the reporting system capture malaria cases? What improvements would strengthen surveillance? | Timeliness, completeness, accuracy; feedback loops; active versus passive detection | Assess surveillance performance and opportunities for strengthening |
| 6. Preparedness and response capacity | Is your zone/woreda/facility prepared for surges or outbreaks? What challenges arise when responding to resurgence? | Staffing and training; infrastructure and supplies; contingency plans; funding and logistics; coordination and governance | Gauge surge preparedness and response bottlenecks |
| 7. Community engagement and awareness | How aware is the community of resurgence and prevention strategies? What are the barriers to participation? | Reach of health education; role of HEWs; religious and traditional leaders; media; sociocultural, economic, and logistical barriers; trust in the health system | Identify levers and barriers for community engagement |
| 8. Environmental and behavioural determinants and care-seeking | Have environmental changes contributed to the resurgence? What behavioural factors contribute to increased cases? | Rainfall/temperature variability; irrigation, construction, deforestation; standing water and drainage; ITN use and care; outdoor sleeping and occupational exposure; treatment adherence; environmental management | Map ecological and behavioural contributors |
|  | Where do people go for diagnosis and treatment? Are there delays in care-seeking? Why? | Public versus private facilities; traditional or religious healers; cost, distance, security; perceptions of severity; alternative explanations | Understand care-seeking pathways and barriers |
| 9. Challenges in malaria control | What challenges do people face in preventing malaria? Are there cultural beliefs or practices that affect prevention or treatment? | Household and community‑level obstacles; hard‑to‑reach groups/areas; specific beliefs or practices | Summarise barriers to effective prevention and care |
| 10. Recommendations | What can be done to improve prevention and treatment? How can the community be more involved? | Resources/support needed from higher levels and partners; coordination between facilities, authorities, and communities; actions to prevent future resurgence | Generate practical, context-specific recommendations |

**Abbreviations:** ITN, insecticide-treated net; IRS, indoor residual spraying; RDT, rapid diagnostic test; HEW, health extension worker; *edir*, traditional mutual aid/funeral association; *ekub*, rotating savings and credit association.

Additional file 3. Key informant interview (KII) guide for malaria experts, coordinators, health professionals, partners, and private sector providers.

| Section/perspective | Main question | Probes | Purpose/notes |
| --- | --- | --- | --- |
| Introduction and background | Please describe your role and responsibilities in malaria work. | Institution/position; years of experience; scope of responsibilities; geographic coverage | Situate the respondent’s perspective (demographics collected separately if needed) |
| 1. Trends in malaria | How would you describe current malaria trends in your area? | Increasing/decreasing/fluctuating/resurgence; timeframe; seasonality; differences by group or location | Establish baseline epidemiological context |
| 2. Linked to of resurgence | What factors are contributing to recent resurgence? Are specific areas or groups more affected? Why? | Socioeconomic, climatic/environmental, health system/service delivery, population movement/displacement factors | Identify perceived linked to and vulnerable groups |
| 3. Vector control | How effective are current vector control measures (e.g. ITNs, IRS)? | Coverage and quality; implementation challenges; community acceptance; insecticide resistance concerns | Assess delivery and performance of vector control |
| 4. Diagnosis and treatment | How accessible and effective are diagnostic and treatment services? | Timeliness; stockouts of RDTs and antimalarials; adherence to national guidelines; perceived treatment failures | Explore availability, quality, and constraints |
| 5. Role of the private sector | What role do private facilities and pharmacies play? | Coordination with the public sector; use of national guidelines; diagnostic and prescribing practices; reporting requirements | Understand contributions, gaps, and regulatory issues |
| 6. Community awareness and behaviour | What is the level of community awareness and adherence to prevention? | ITN use and care; acceptance of IRS; cultural beliefs; barriers to uptake | Examine behavioural and sociocultural determinants |
| 7. Impact of emergencies and context | How have public health emergencies (e.g. COVID‑19) affected malaria control? Are there political, security, or economic factors influencing the programme? | Surveillance; case management; vector control; resource shifts; access constraints | Document contextual shocks and system fragility |
| 8. Environmental determinants | What environmental changes are shaping current trends? | Rainfall/temperature patterns; land use, irrigation, construction; urbanisation | Explore ecological determinants and mitigation |
| 9. Surveillance and policy | How effective is malaria surveillance for detection and response? Have policy or strategic changes influenced trends? | Reporting gaps; data quality; use of data for decisions; active versus passive case detection; implementation/funding effects | Assess surveillance function and policy context |
| 10. Recommendations | What actions would most strengthen malaria control in this area? | Urgent needs; coordination across levels/sectors; role of partners and private sector | Elicit concrete, feasible, and time-bound recommendations |

**Abbreviations:** ITN, insecticide-treated net; IRS, indoor residual spraying; RDT, rapid diagnostic test; COVID-19, coronavirus disease 2019

Additional file 4: In-depth interview (IDI) guide for community leaders, volunteers, and Associations

| Section/topic | Main question | Probes | Purpose/notes |
| --- | --- | --- | --- |
| Introduction | — | Introduce interviewer; explain the study purpose; emphasise confidentiality, anonymity, voluntary participation, and the right to stop at any time; explain audio recording and expected duration; obtain informed consent | Set context, build rapport, and ensure ethical conduct |
| 1. Community experience of malaria | How has malaria affected your community in recent years? | Perceived trends (increases, decreases, fluctuations); who is most affected (children, pregnant women, men, women, specific occupations or locations); impacts on health, work, school attendance, and household income | Understand perceived trends and socioeconomic impacts of malaria at the community level |
| 2. Reasons for increased cases | What do you think are the main reasons for any increase in malaria cases? | Environmental changes (deforestation, farming practices, irrigation schemes, construction sites); presence of standing water (canals, ponds, pits, tanks); shortages or poor quality of bed nets or medicines; behaviours that increase risk (sleeping outdoors, not using nets, delayed care‑seeking); population movement or displacement | Identify local explanations for malaria resurgence across environmental, health system, and behavioural domains |
| 3. Prevention tools (ITNs, IRS) | Are tools such as insecticide‑treated nets (ITNs) and indoor residual spraying (IRS) being used effectively in your community? | Household ownership and sufficiency of ITNs; correct and consistent use (who uses nets, when, and how); condition of nets (torn, old, or repurposed); IRS coverage (which areas or households are sprayed, which are missed, and why); acceptance or refusal of IRS; reported side‑effects or complaints | Explore access to and correct use of prevention tools, and barriers to their effective use |
| 4. Knowledge and information | How well informed are people in your community about malaria prevention and treatment? | Main information sources (HEWs, health professionals, radio, religious leaders, schools, neighbours, social media); common messages remembered; misconceptions or myths about malaria causes, transmission, or treatment; trust in health workers, HEWs, and government programme; rumours that affect prevention or care‑seeking | Assess malaria‑related knowledge, information sources, misconceptions, and trust in the health system |
| 5. Environmental and seasonal factors | How do weather and environmental conditions affect malaria here? | Perceived links between rainfall and malaria; presence of standing water after rain; changes in rivers, ponds, irrigation canals, and water tanks; impacts of new farming schemes, dams, or construction projects; differences between rainy and dry seasons in terms of mosquito density and malaria cases | Investigate perceived environmental and seasonal contributions to malaria risk |
| 6. Access to health services | What challenges do people face when they need diagnosis or treatment for malaria? | Usual care‑seeking pathways (public health facilities, private clinics, pharmacies, traditional or religious healers); distance and travel time to facilities; transport availability and cost; user fees and other costs; opening hours and waiting times; stockouts of tests or drugs; experiences in conflict‑affected or insecure areas (roadblocks, closed facilities, displaced health workers) | Identify barriers and facilitators to timely, appropriate diagnosis and treatment |
| 7. Community activities and associations | How do community groups or volunteers contribute to preventing and controlling malaria? | Roles of *edir*, *ekub*, women’s groups, youth groups, and other associations; examples of activities (environmental clean‑up, health education, supporting vulnerable households); perceived successes and challenges; support received from government, NGOs, or health facilities; suggestions for how these groups could be more involved | Understand existing community‑driven initiatives and opportunities to strengthen them |
| 8. Behavioural change | Have people’s behaviours towards malaria prevention and treatment changed in recent years? If so, how? | Changes in perceived susceptibility and severity of malaria; changes in ITN ownership and use, IRS acceptance, or environmental management; delays in seeking care or increased promptness; motivations for adopting or rejecting preventive behaviours; influence of religious or traditional beliefs; examples of individuals or groups who changed behaviour and why | Examine changes in attitudes and practices over time, and identify levers for behaviour change |
| 9. Support from government and NGOs | How have government or NGO efforts addressed malaria in your community? | Recent malaria-related activities (ITN distribution, IRS campaigns, health education, environmental management); perceived effectiveness and gaps; coordination between government, NGOs, and community structures; how support could be improved to better meet community needs | Assess alignment of external support with community priorities and needs |

**Abbreviations:** HEW, health extension worker; ITN, insecticide-treated net; IRS, indoor residual spraying; NGO, non-governmental organisation; *edir*, traditional mutual aid/funeral association; *ekub*, rotating savings and credit association

Additional file 5. COREQ 32-item checklist, and where each item is reported in the manuscript

| No. | Domain | Item | Where reported in the manuscript |
| --- | --- | --- | --- |
| 1 | Domain 1: Research team and reflexivity | Interviewer/facilitator | Methods – Data collection tools and procedures |
| 2 |  | Credentials | Title page; Methods – Reflexivity |
| 3 |  | Occupation | Title page; Methods – Reflexivity |
| 4 |  | Gender | Methods – Data collection tools and procedures |
| 5 |  | Experience and training | Methods – Reflexivity |
| 6 |  | Relationship established | Methods – Study population, sampling and recruitment |
| 7 |  | Participant knowledge of the interviewer | Methods – Study population, sampling and recruitment; Additional files 1–3 (introduction sections) |
| 8 |  | Interviewer characteristics | Methods – Reflexivity |
| 9 | Domain 2: Study design | Methodological orientation | Methods – Study design and period |
| 10 |  | Sampling | Methods – Study population, sampling and recruitment |
| 11 |  | Method of approach | Methods – Study population, sampling and recruitment |
| 12 |  | Sample size | Methods – Study population, sampling and recruitment |
| 13 |  | Non‑participation | Methods – Study population, sampling and recruitment |
| 14 |  | Setting of data collection | Methods – Data collection tools and procedures |
| 15 |  | Presence of non-participants | Methods – Data collection tools and procedures |
| 16 |  | Description of sample | Results – Sociodemographic characteristics of participants; Table 1 |
| 17 | Domain 3: Analysis and findings | Interview guide | Methods – Data collection tools and procedures; Additional files 1–3 |
| 18 |  | Repeat interviews | Methods – Data collection tools and procedures |
| 19 |  | Audio/visual recording | Methods – Data recording and management |
| 20 |  | Field notes | Methods – Data collection tools and procedures |
| 21 |  | Duration | Methods – Data collection tools and procedures |
| 22 |  | Data saturation | Methods – Study population, sampling and recruitment |
| 23 |  | Transcripts returned | Not performed (not applicable) |
| 24 |  | Number of data coders | Methods – Data analysis |
| 25 |  | Description of coding tree | Methods – Data analysis (codebook development and concept mapping) |
| 26 |  | Derivation of themes | Methods – Data analysis |
| 27 |  | Software | Methods – Data analysis (ATLAS.ti version 9.0) |
| 28 |  | Participant checking | Not performed (not applicable) |
| 29 |  | Quotations presented | Results (all themes); Table 2; Additional files-5 |
| 30 |  | Data and findings are consistent | Addressed throughout Results and Discussion |
| 31 |  | Clarity of major themes | Results – Themes (main headings) |
| 32 |  | Clarity of minor themes | Results – Subheadings and within-text elaboration |

Additional file 6. Thematic map of factors contributing to malaria resurgence in the Amhara Region

| Theme | Sub‑theme | Example codes | Exemplar quotation(s) |
| --- | --- | --- | --- |
| Environmental and climate drivers | Irregular rainfall and temperature change | Climate change, prolonged rainfall, stagnant water, and enhanced mosquito breeding | “We’ve experienced unusual rainfall over the past three years, leading to widespread stagnant water everywhere—ideal breeding grounds for mosquitoes.” (KII–zonal malaria expert, male, 43 years) |
|  | Development projects and landscape modification | Unregulated construction; irrigation schemes; deforestation; human-made breeding sites; migrant workers | “Irrigation schemes contribute to mosquito proliferation and attract non-immune migrant workers, thereby increasing the spread of malaria.” (KII–regional malaria programme coordinator, male, 40 years) |
| Health system gaps | Drug and supply stockouts | Supply chain failure; stockouts of Coartem, primaquine, RDTs; ineffective case management; reliance on the private sector | “Coartem has been out of stock for four to five days every month… chloroquine and primaquine were unavailable for six months.” (KII–health officer, female, 34 years) |
|  | Weakened vector control | Inconsistent IRS; delayed ITN distribution; lack of larvicides; outdated strategies; worn‑out nets | “Many families still use worn‑out mosquito nets, and the spraying programme hasn’t reached all villages for the last two to three years.” (IDI–community volunteer, male, 46 years) |
|  | Fragile surveillance and service delivery | Passive surveillance; under‑reporting; diagnostic gaps; reduced role of HEWs; unregulated private clinics | “The health extension programme’s contribution to malaria prevention has dropped to 20–30%, far below the expected 70%.” (FGD–expert, mixed gender) |
| Community engagement and perceptions | Low risk perception and delayed care‑seeking | Normalisation of malaria; low perceived susceptibility; delays in treatment; reliance on traditional healers; misattribution of symptoms | “Communities often wait until they are severe because travelling is difficult, and even health posts are closed.” (IDI–community leader, male, 40 years) “Some believe fever is due to spiritual causes or other illnesses, not malaria…” (KII–health officer, female, 48 years) |
|  | Poor adherence and misconceptions | ITN misuse/repurposing; misconceptions about causes; discomfort with nets; doubts about effectiveness | “Bed nets are used for storage, as a garden fence, or to make gemed (rope)… not for prevention.” (KII–woreda malaria officer, male, 40 years) |
|  | Weak cues to action and low self‑efficacy | Inconsistent health education; lack of reminders; feelings of helplessness; distrust in the health system | “No one reminds us to drain stagnant water.” (IDI–community leader, male, 44 years) “Mosquitoes everywhere overwhelm me; I feel helpless, unable to fight them off.” (IDI–community leader, male, 41 years) |
| External drivers | Conflict and political instability | Disruption of services; displacement of health workers; supply chain blockades; budget diversion; reduced partner engagement | “Ongoing conflict has displaced communities and diverted health budgets from malaria control to emergency responses.” (KII–zonal malaria officer, male, 46 years) |
|  | Impact of COVID‑19 | Deprioritisation of malaria, reduced surveillance; disruption of routine services | “During the pandemic, malaria was deprioritised, leading to a reduction in surveillance and control activities.” (KII–woreda malaria officer, male, 38 years) |
| Emerging biological threats | Perceived drug and insecticide resistance | Treatment failure; recurring infections; ineffective IRS | “Patients return with recurring infections despite completing treatment.” (KII–woreda malaria officer, male, 32 years) “Mosquito density didn’t drop after IRS, implying resistance.” (KII–regional malaria expert, male, 42 years) |
|  | Concerns about invasive vectors | *Anopheles stephensi*; urban malaria transmission | “The invasive mosquito species *Anopheles stephensi*… is driving urban malaria transmission.” (FGD–experts, mixed gender) |

**Abbreviations:** HEW, health extension worker; IRS, indoor residual spraying; ITN, insecticide-treated net; KII, key informant interview; IDI, in-depth interview; RDT, rapid diagnostic test; FGD, focus group discussion; COVID‑19, coronavirus disease 2019.

Additional file 7. Application of the Health Belief Model to community perceptions and behaviours regarding malaria resurgence

| HBM construct | Definition* | Illustrative quotations (data source) |
| --- | --- | --- |
| Perceived susceptibility | Belief about the likelihood of getting malaria | *“I know malaria is around, but I don’t think I can get it. I keep my house clean, so I feel safe.”* (IDI, community leader, female, 42 years)*, “Malaria is seasonal; we don’t think it will affect us severely.”* (IDI, village health leader, male, 44 years) |
| Perceived severity | Belief about the seriousness of malaria and its consequences | “*People talk about malaria like it’s just a fever, but I’ve seen people die from it.”* (IDI, community volunteer leader, male, 39 years), *Malaria was often perceived by participants as a non-fatal, chronic, normal part of life.* |
| Perceived benefits | Belief in the effectiveness of recommended actions (e.g., ITNs, IRS) to reduce risk or severity | “*Indoor residual spray chemicals are ineffective; mosquito density did not drop after IRS.”* (FGD, experts, mixed gender), "*After they sprayed, we saw no change. The mosquitoes were just as wicked. We think the chemical no longer works*." (IDI, community volunteer, male, 37 years) |
| Perceived barriers | Belief about the tangible and psychological costs of taking preventive action | “*Public health facilities run out of drugs, pushing patients to costly private clinics.” (FGD, clinical, mixed gender), “Communities often wait until they are severe because travelling is difficult, and even health posts are closed*.” (IDI, community leader, male, 40 years) “*Some households still resist using bed nets, citing discomfort and cultural beliefs*.” (IDI, village health elder, male, 42 years) |
| Cues to action | Factors that trigger or prompt individuals to take preventive or treatment actions | “*No one reminds us to drain stagnant wate*r.” (IDI, community leader, male, 44 years). “*There are no mass media campaigns to reinforce prevention messages*.” (FGD, clinical, mixed gender) |
| Self-efficacy | Confidence in one’s ability to prevent malaria or seek care | “*Mosquitoes everywhere overwhelm me; I feel helpless, unable to fight them off*.” (IDI, community leader, male, 41 years). “*Communities lack ownership of environmental management.*” (KII, regional malaria expert, male, 48 years) |
| *Definitions adapted from Champion and Skinner [19]. | | |
